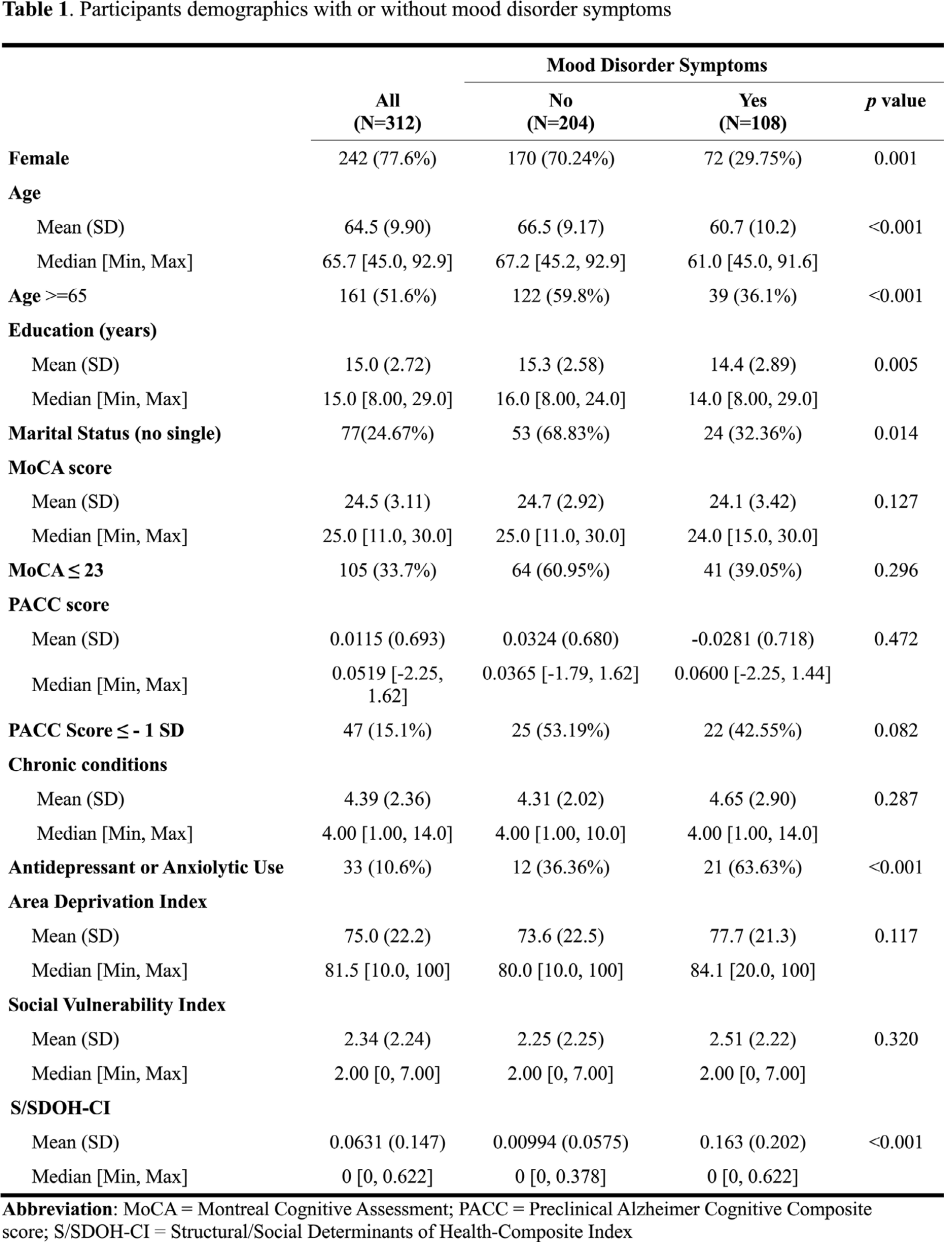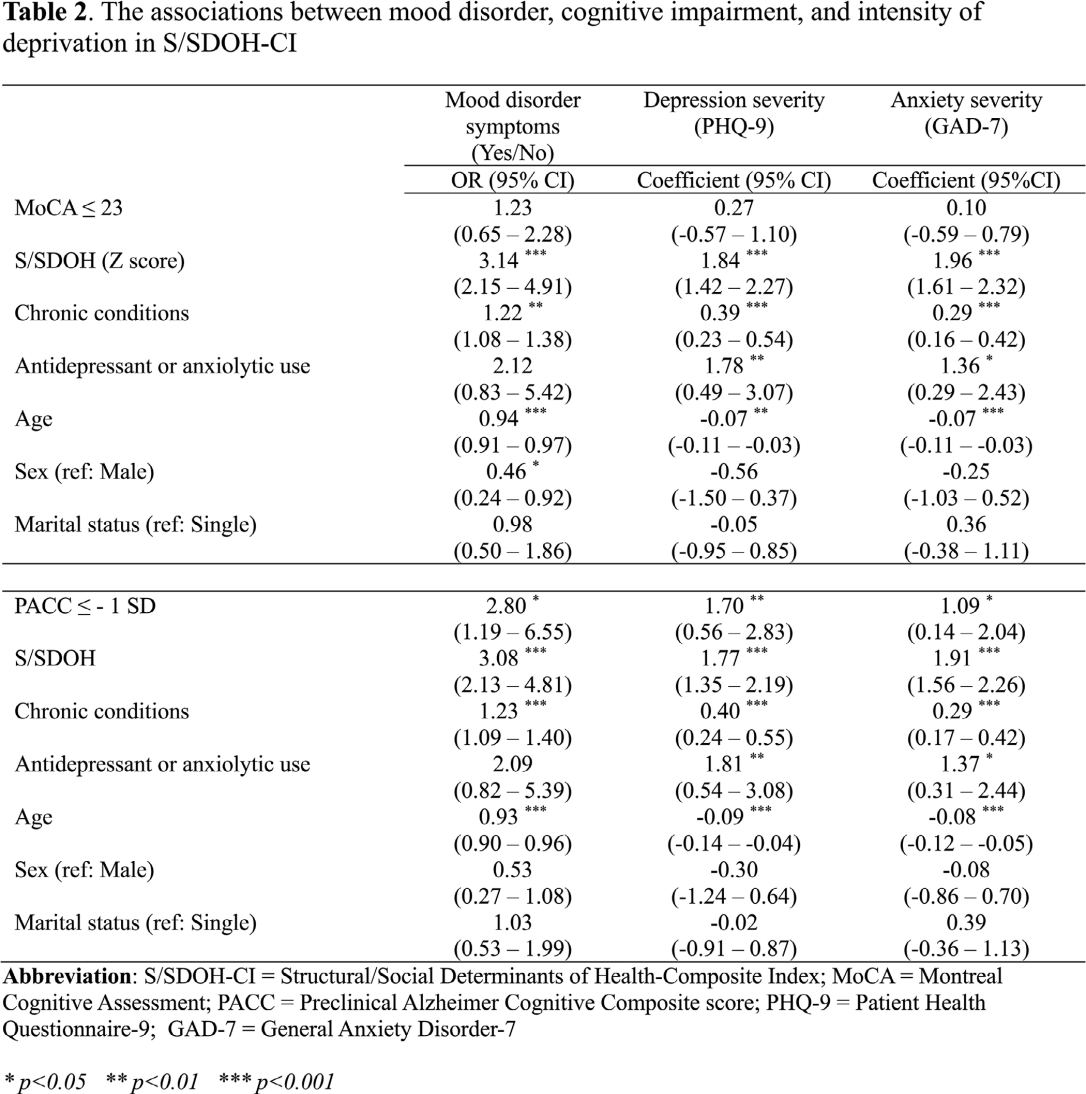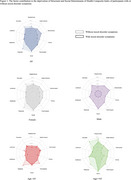# The Association between Cognitive Impairment, Structural and Social Determinants of Health, and Mood Disorder among Black Adults in the Greater St. Louis Metropolitan Area

**DOI:** 10.1002/alz70860_104347

**Published:** 2025-12-23

**Authors:** Yiqi Zhu, Jean‐Francois Trani, Alexis I. B. Walker, Jonathan Williams, Ganesh M. Babulal

**Affiliations:** ^1^ Washington University School of Medicine, Saint Louis, MO, USA; ^2^ Washington University, St. Louis, MO, USA; ^3^ Natiobal conservatory of Arts and Crafts, Paris, NA, France; ^4^ Washington University School of Medicine, St.Louis, MO, USA; ^5^ University of Johannesburg, Johannesburg, Gauteng Province, South Africa; ^6^ Knight Alzheimer Disease Research Center, St. Louis, MO, USA

## Abstract

**Background:**

Black middle‐aged and older adults face a significantly higher risk of mood disorders and cognitive impairment compared to non‐Hispanic Whites. Prior evidence underscores that the structural and social determinants of health (S/SDOH) are estimated to contribute to 60‐80% of all health outcomes. However, evidence examining the intersection of S/SDOH and depression and anxiety remains fragmented, often focusing on limited measures like income or socioeconomic status or using aggregated measures at the census tract. This study utilized a new S/SDOH measure based on the National Institute on Aging Health Disparities Research Framework using individual and household level data to examine the relationship between mood disorders (depression and anxiety), cognitive impairment, and S/SDOH.

**Methods:**

Baseline data from Black participants aged 45 and older (*n* = 312) in the Greater St. Louis Metropolitan region were examined. A S/SDOH composite index (S/SDOH‐CI) was constructed to measure participants' deprivation and unique risk factors. Depression and anxiety symptoms were measured using the Patient Health Questionnaire (PHQ‐9) and the Generalized Anxiety Disorder 7 item (GAD‐7). Cognitive impairment was assessed using the Montreal Cognitive Assessment (MoCA) and a preclinical Alzheimer's cognitive composite (PACC) score.

**Results:**

Among 312 cognitively normal participants, the average age was 64.5 (SD = 9.9), and the average education level was 15 years (SD = 2.82). 77.6% were female, and 108 (35%) had at least mild mood disorder symptoms. Logistic regression analysis indicated that a one SD increase in the burden of deprivation was associated with 3.12 times the odds of having any symptoms (95% CI: 2.13–4.92). Furthermore, contribution to S/SDOH‐CI revealed hardship and daily stressors (17%), and discrimination (11%) were the primary factors exacerbating mood disorders. These surpassed the contribution of education (10.67% [years and quality]), particularly among Black men, highlighting racism as a formidable driver of health inequities and lending additional evidence that there may be diminishing returns on human capital investments among Black men.

**Conclusion:**

These findings emphasize the need for medical advancements to progress together with social justice initiatives. The use of the S/SDOH‐CI uncovered within‐group differences and identified unique risk and resilience factors for mood disorders in Black older adults.